# Multi-stage treatment for malunion and avascular necrosis of the femoral head following reverse oblique pertrochanteric fracture: A case report and literature review

**DOI:** 10.1016/j.tcr.2022.100684

**Published:** 2022-08-01

**Authors:** Przemyslaw T. Paradowski, Kamil Sadzikowski, Piotr Majewski, Marek Szczepaniec

**Affiliations:** a Department of Orthopaedics and Traumatology, Jan Biziel University Hospital, Ujejskiego 75, PL-85-168 Bydgoszcz, Poland; b Department of Surgical and Perioperative Sciences, Division of Orthopedics, Sunderby Research Unit, Umeå University, Sunderby Central Hospital of Norrbotten, SE-971 80 Luleå, Sweden; c Clinical Epidemiology Unit, Orthopedics, Department of Clinical Sciences Lund, Lund University, SE-221 85 Lund, Sweden

**Keywords:** Induced membrane Masquelet technique, Bone defect, Reverse oblique intertrochanteric fracture, Osteonecrosis, Femoral head

## Abstract

Femoral reverse oblique intertrochanteric fractures are successfully treated using intramedullary devices. Poor fracture reduction and the use of the inaproppriate implant can lead to implant failure and subsequent malunion or non-union.

We present the case of a 21-year-old polytrauma female who, among other injuries, sustained a reverse oblique intertrochanteric fracture that was primarily operated on with the dynamic hip screw. After implant failure the patient developed malunion with bone defect and avascular necrosis of the femoral head. Successful salvage of the bone stock in the inter- and subtrochanteric region was achieved using the two-stage induced membrane technique. The uncemented total hip arthroplasty was performed to address the osteonecrosis of the femoral head.

## Introduction

Trochanteric fractures among young adults are generally caused by high-energy traumas [1]. Most of these fractures belong to type 31-A2 and A3 according to the AO/OTA classification [2] and are regarded unstable. A3 fractures have unique anatomic and mechanical characteristics, including a fracture line exiting the lateral femoral cortex distal to the vastus ridge of the greater trochanter [3], [4] and thus they are referred to as reverse obliquity fractures.

Treatment of reverse oblique and transverse intertrochanteric fractures is challenging due to the shear forces leading to the medialization of the femoral shaft [5]. Since the force from the femoral head to shaft is distributed predominantly through the calcar femorale [6], [7], pertrochanteric fractures with compromised medial calcar need to be appropriately reduced and stabilized with a device that is able to transfer medial compressive loads [5], [8]. These conditions are fulfilled better by an intramedullary nail that is inserted closer to the force vector line and has a shorter lever arm than a sliding hip screw located more laterally [8]. In fact, most authors have reported that in unstable trochanteric fractures, intramedullary nails achieve fewer complications than extramedulary implants [3], [4], [9], [10], [11]. With a failure rate up to 56 %, the sliding hip screw is regarded as not suitable for management of AO/OTA 31-A3 fractures [12].

Fixation failure due to the use of an inaproppriate implant, suboptimal fracture reduction [13] and residual displacement [14] with the lack of medial cortical support [15] can lead to severe persistent pain, increased length of hospital stay, decreased quality of life and functional status. The most common complications include osteonecrosis of the femoral head (ONFH), fracture non-union or malunion and screw cut-out or implant breakdown [16] and they constitute indications for reoperation. Salvage osteosynthesis with or without bone grafting may be preferable for patients with long life expectancy and enough proximal femoral quality for fixation [17]. However, in subjects with ONFH, regardless of their age, the conversion hip arthroplasty may be a more viable treatment option [18], [19].

Since the revision of intramedullary fixation imposes serious technical challenges, detailed preoperative planning is required. The most effective treatment of proximal femoral non-unions and malunions with bone loss includes the removal of any existent implants, grafting of bone defects, correction of deformities and, finally, stable osteosynthesis [20].

It has commonly been assumed that small to mid-size bone defects should be managed with the use of non-vascularized bone grafts and that larger defects need to be vascularized. There is, however, no clear indication how long the graft must be to require vascularization [21]. Since a flexible and individualised approach is demanded, some alternative reconstructive techniques can also be used. The Masquelet technique is a two-stage procedure that involves the formation of a fibrous membrane induced with a polymethylmethacrylate (PMMA) spacer prior to filling the defect cavity with cancellous graft material [22], [23]. The method has been found to be feasible in patients with large posttraumatic defects or infected non-unions in long bones [22], [24], [25], [26], [27], [28].

In this report, we present the case of a patient with a malunion in the proximal femur following the failure of AO/OTA 31-A3 fracture osteosynthesis. We used a modified Masquelet technique to reconstruct a four-centimeter bone defect.

## Case report

**Fig. 1:**
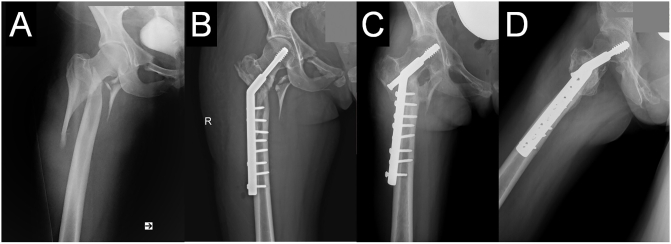
Pre-operative radiograph showing the reverse oblique intertrochanteric AO/OTA 31-A3 fracture (A), postoperative radiograph of the right hip 2 weeks after the initial operation (B) and a radiograph made nine months after the first surgery with visible osteonecrosis of the femoral head and cutout of the lag screw (C, D).

The patient was a 21-year-old amateur rock climber who survived a free fall from a height of 18 m into a cave in September 2018. She fell off the end of an unknotted rope and hit the cave climbing instructor who was severeal meters below, which attenuated her fall and saved her life. The climber sustained a serious damage to the right side. After two hours she was brought back up to the surface by the rescuers and taken by helicopter to the regional level 1 trauma center. The patient was in a stable condition. She sustained a comminute reverse oblique pertrochanteric femoral fracture (AO/OTA 31-A3) with comminution of both the posteromedial and posterolateral cortices and medial dislocation of the distal femur fragment. These injuries indicated a highly unstable fracture system (Fig. 1A).

In addition, the patient had burst fractures of the thoracic vertebra Th12 and lumbar vertebra L1 with significant vertebral body comminution but without neurological deficits, as well as a type 1 lateral compression pelvis fracture (LC1) with an undisplaced fracture of the sacrum and an ipsilateral fracture of the os pubis.

### Spine operation

Posterior fracture reduction, transpedicular correction and trisegmental stabilization between Th10 and L2 were performed five days after admission. The patient had no external immobilization during the postoperative period, and was allowed to sit up in bed and to walk as soon as her clinical condition permitted. No complications were observed.

The pelvis fracture was treated non-operatively.

### Primary hip operation

The hip operation was performed under general anesthesia, with the patient in a supine position on an operating table with traction five days after the accident, at the same operative session as the spine surgery. The patient was scheduled for internal fixation by means of a dynamic hip screw (DHS) with an eight-hole DHS plate and eight conventional 4.5 mm bicortical screws. The fracture was manually reduced and stabilized under image intensifier control.

Postoperative anteroposterior radiographs showed suboptimal reduction of the fracture. Even though the position of the lag screw in the femoral head was inferior (with Parker's ratio of 43 [29]), the distal femoral fragment together with side-plate was displaced medially in relation to the comminutively fractured greater trochanter. These findings could be consistent with the observed persistent axial, and rotational instability [16].

Postoperatively, parenteral antibiotics were scheduled for three days. Since the postoperative wound was superficially infected with enterococcus faecalis and *serratia marcescens*, the antimicrobial therapy with amoxiccilin with clavulonic acid was continued for additional four weeks. The patient left the hospital with wound care instructions and deep venous thrombosis prophylaxis with dalteparine for three months. After discharging from the orthopedic ward, the patient was admitted to the rehabilitation center for an intensive 6-week full-day inpatient exercise program, which she continued at home. She was not allowed to bear any weight on the injured leg for three months, then was allowed only to toe touch weight bearing. The patient was successively followed up at the regional university orthopedic trauma center.

The patient developed moderate to severe pain in the right hip that worsened with time and became more constant. No additional injury occurred. She presented limitation of movement in all directions. The follow-up antero-posterior radiography and complementary examination with computed tomography (CT) from July 2019 revealed a collapse of the fracture with medial migration of the distal femoral fragment, malunion as well as avascular necrosis of the femoral head, consistent with Ficat stage III [30], and an incipient central cutout of the lag screw (Fig. 1C).

The patient was then scheduled for a surgery to remove the proximal part of the femur and to carry out modular reconstruction with a megaprosthesis.

The patient asked questions about alternative treatment options and potential prognosis. She expressed a wish to seek a second opinion in another hospital and, consequently, sought help in our department.

The physical *examination* showed a lower right leg shortening of 4 cm. All hip joint movements were limited with a marked loss of hip flexion (40 degrees), abduction (10 degrees) and rotation (15 degrees externally and 5 degrees internally). The x-ray that was made a year after the injury showed Ficat grade IV with decalcification, flattening of the femoral head, loss of the articular cartilage and development of acetabular osteophytes (picture not shown) [30].

We identified several problems that had to be addressed, including bone malunion with a leg length discrepancy, deformity of proximal femur and ONFH. Taking into account all factors that allow to predict bone healing, such as the patient's young age and long life expectancy, her high functional level, good quality of bone, salvage osteosynthesis to attain a bone union, followed by conversion total hip arthroplasty (THA) to restore the hip joint function, appeared to be the treatment of choice.

### Masquelet operation

The operation took place in October 2019, 13 months after the primary injury. It was performed with the patient in general anesthesia, in the supine position on a fracture extension table. A lateral incision was carried out through the patient's old scar. The abundant scar tissue was removed to expose the deformed pertrochanteric area and dislocated DHS plate. After the sliding hip screw was successfully withdrawn and the trochanteric stabilizing plate hardware removed, a radical resection of the dead bone was made.

Fractured bone surfaces were then freshened with repermeabilization of the medullary canal. The length of the bone defect was 4 cm. The residual cavity after lag screw removal was then filled with cancellous bone grafts. Restoration of the femoral length was obtained with traction. The diameter of the nail was calculated preoperatively with regard to thick cortex and narrow medullary canal. The femoral canal was enlarged by reaming, beginning with hand reamers of 7.0, 7.5 and 8.0 mm. Then a flexible shaft with an 8.5 mm front-cutting reamer was applied and reaming progressed in 0.5 mm increments up to 10 mm. A long double proximal femoral nail with the length of 370 mm, diameter of 8.0 mm and neck-shaft angle of 125 degrees (Medgal, Księżyno, Poland) was inserted. A cephalic lag screw was positioned in the *inferior* 1/3 area (in anteroposterior view) of the femoral head. The tip-apex distance was not considered crucial, since the purpose of stabilization was only to prevent a/the secondary hip dislocation. The bone defect was then filled with a cylindrical polymethylmethacrylate bone cement spacer (Refobacin Plus, Biomet, Valence, France), covering bony ends, which also augmented the stability (Fig. 3A). The soft tissue envelope was then repaired without tension over the spacer and the wound was closed.

**Fig. 2:**
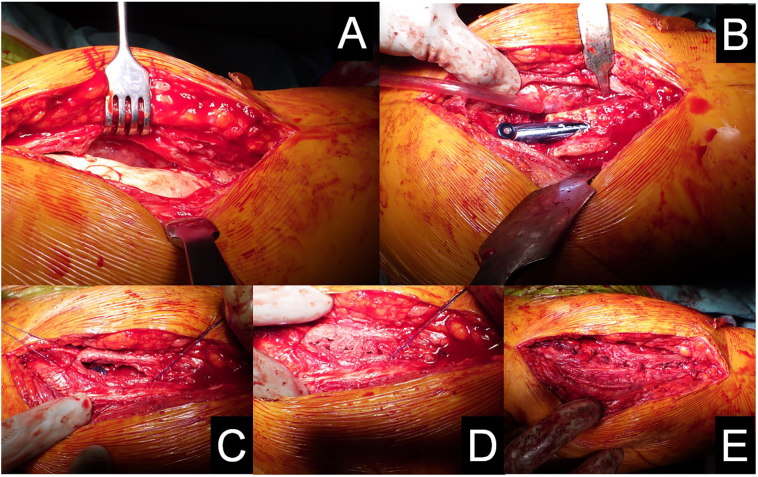
Masquelet procedure at stage two. Bone defect filled with PMMA cement spacer (A), bone defect after removing of spacer with the intramedullary nail exposed (B), bone graft within the induced membrane (C, D) and, finally, the membrane closed with Vicryl sutures (E).

The cement spacer was left in place for 5 weeks. During the second operation (November 2019), a careful sharp dissection of the newly formed biological membrane was performed to remove the cement spacer (Fig. 2A and B). The defect volume was calculated according to the formula V = πhr^2^ [31] and it was 26 cm^3^ when the volume of intramedullary nail was counted out. To fill the bone defect within the membrane, we utilized a combination of an autologous graft harvested from the ipsilateral iliac crest and a morselized cancellous allograft provided by the local bone bank, mixed in a ratio of 1:3 (Fig. 2C and D). No platelet-rich plasma or growth factors were used for graft enrichment. The membrane was then closed with absorbable Vicryl 0 sutures (Fig. 2E).

After stage-one and stage-two operations, the patient was given three doses of intravenous prophylactic antibiotics, 2 g of cefazolin (Cefazolin, MIP Pharma GmbH, Blieskastel, Germany). Postoperatively, non-weight-bearing walking with crutches was recommended for six weeks. Partial weight bearing was started from week 6 and continued until the definitive operation.

**Fig. 3:**
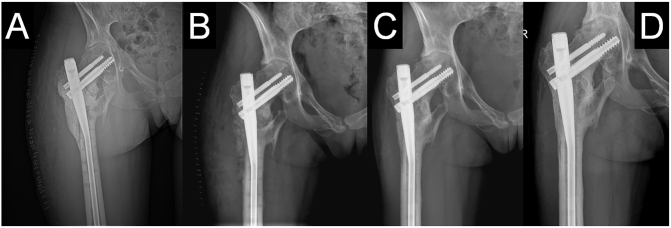
Anteroposterior (AP) pelvic radiographs showing bone defect filled with PMMA cement spacer after stage one operation (A) and graft integration immediately (B), 3 months (B), 6 months (C) and 11 months (D) after stage two Masquelet operation.

The patient was followed up every three months with both the clinical and radiological assessments (Fig. 3B–D). Bone healing and union of the hip fracture was achieved at 9 months following the Masquelet procedure. No infection was observed. Besides, we observed a progressing deformation of the femoral head due to ONFH. Postoperative *hip* motion was poor, with 40 *degrees of flexion*, 20 *degrees of* abduction, 20 *degrees of* external rotation, and less than 10 *degrees of internal rotation.* The patient was consequently scheduled for a primary THA.

## THA

The operation was performed under general anesthesia in the lateral decubitus position. The direct lateral approach according to Gammer [32] was used. The proximal aspect of the intramedullary nail was exposed in the usual fashion. After removal of the lag screw and derotatory pin, the locking screws were removed from the separate incision distally in the femur. The nail was extracted without any troubles.

The deformed femoral head was removed, revealing an enlarged acetabulum filled with osseous debris and cartilage splints. The acetabulum was then reamed in a concentric fashion in 2 mm increments to remove all remaining cartilage.

The last reamer used was 1 mm smaller than the corresponding cup size, allowing a press-fit insertion of the cup. A 50-mm Continuum trabecular metal cup combined with a neutral crosslinked polyethylene insert (Zimmer, Warsaw, USA) were implanted at the anatomic position. One screw was used to augment the primary stability of the cup.

To deal with the proximal femoral deformities and the narrow diaphyseal canal, as well as to achieve a stable stem anchorage distally to the restored bone in proximal diaphysis, a modular stem was chosen to be implanted. Preparation of the medullary canal was undertaken with tapered reamers and broaches of gradually increased size until the outlined diameter of 14 mm was achieved. Then conical and triangular reaming of the metaphysis was performed to prepare space for a proximal sleeve. A sufficient diaphyseal anchorage was provided with a conically shaped Revitan Straight implant system of 140 mm in length (Zimmer, Warsaw, USA), combined with a proximal spout component of 55 mm in height. After all components were placed into position, stability and range of motion were assessed. The hip was finally reduced with a 32-mm Biolox delta ceramic head.

The patient received three doses of prophylactic intravenous antibiotic, 2 g of cefazolin (Cefazolin, MIP Pharma GmbH, Blieskastel, Germany) and was given low molecular weight heparin and 40 mg of enoxaparin sodium administered by subcutaneous injection (Clexane/Lovenox, Sanofi-Aventis) once daily for six weeks.

**Fig. 4:**
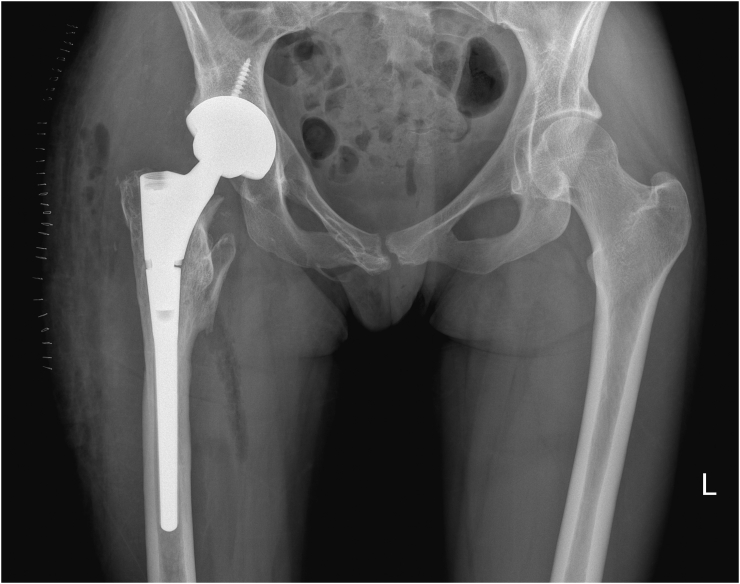
Standing AP pelvic radiograph taken after total hip arthroplasty.

Unrestricted weight-bearing was allowed. Postoperative radiographs showed no leg length inequality and symmetrical vertical and horizontal centers of rotation (Fig. 4). The acetabular inclination of the implant was 43 degrees and anteversion 17 degrees.

The patient was followed up at regular intervals, three, six and 12 months after the operation. At every follow-up appointment, she underwent a physical examination and completed the Hip dysfunction and Osteoarthritis Outcome Score (*HOOS*) forms.

Hip flexion improved from 40 degrees before to over 90 degrees at 6 months after THR. No lower limb-length discrepancy was observed. The gait with slightly decreased single support time for the right limb and shortened step length for the contralateral limb was observed at the 3-month follow-up. No limp was noted at the 6-month follow-up. The outcome scores in all HOOS domains improved between follow-up examinations (Table 1).

**Table 1:** The outcome in the Hip dysfunction and Osteoarthritis Outcome Score (*HOOS*) at 3-, 6- and 12-month follow-up.

Time from THA	HOOS domains				
	Pain	Symptoms	ADL	Sport/Rec.	QOL
3 months	72.5	80	55.9	31.3	43.8
6 months	85	65	81	62.5	50
12 months	90	90	86.8	56.3	75

Abbreviations: THA total hip arthroplasty; ADL activities of daily living; QOL Quality of life.

## Discussion

We report a case of a successfully treated malunion of the proximal femur following improperly treated reverse oblique intertrochanteric fracture. We performed a multi-stage treatment consisting of reconstruction of the proximal femur with the use of the technique described by Masquelet, and THA, as a definitive solution for the ONFH.

Bone healing complications of intertrochanteric fractures are uncommon due to the excellent blood supply and good cancellous bone quality in the proximal femur [33]. Non-union, malunion or early failure of fracture fixation might occur, however, due to delayed treatment, unfavorable fracture patterns, poor bone quality or suboptimal internal fixation. Some of these inauspicious factors were present, in fact, in our patient.

The patient was operated on five days after admission, which was not according to the recommended guidelines [34], [35]. However, an acceptable delay time has not yet been established and so far there is no evidence that an operation made four days beyond the recommended time frame might predispose young and generally healthy subjects to complications with bone healing.

The initial operation was performed with a DHS plate. This technique has been proved to be a highly successful method for fixation of stable inter-trochanteric fractures. However, since AO/OTA 31-A3 fractures are biomechanically unstable by nature [12], a DHS plate, especially when applied without an additional trochanteric stabilizing plate (TSP), is commonly regarded as an inappropriate method in the treatment of such injuries [12], [36], even if literature sources are not explicit [37]. Treating highly instable intertrochanteric fractures with a DHS plate may, in particular, lead to a loss of reduction, non-union, and/or malunion with a varus deformity of the femoral neck, marked shortening of the affected limb or a screw cutout [38].

It is unclear to us why that method was used. The surgeons performing the initial operation should have considered using a reamed intramedullary nail as stabilization even if the intramedullary canal was narrow. Our experience shows that a proper nail is available within three days, so a turnaround delay cannot be used as an excuse for the surgeon's choice.

The suboptimal reduction of the fracture with radiological signs of instability was already seen on the first postoperative radiographs (Fig. 1B). These early signs of failure were an indication for re-osteosynthesis. However, the revision surgery had not been made before the patient consulted our department.

The improper selection of implant associated with insufficient fixation and, consequently, inadequate mechanical stability resulted in two problems: a mechanical problem related to load bearing, and a biological one connected with disturbed vascularization of the femoral head. The former led to a malunion in the intertrochanteric region, the latter to the ONFH.

Before she came to our hospital, the patient was subjected to the operation with the use of resection megaprosthesis. We suppose that such an approach was suggested with the intention to perform only one definitive operation and make the whole treatment shorter. In our opinion, the resection of the proximal femur, albeit it is a relatively simple procedure, was unreasonable.

Management of an extensive proximal segmental femoral bone loss remains controversial. The most established indication for the use of a megaprosthesis is a reconstruction after resection of primary malignant bone tumors [39]. In addition, it has been advocated that magaprostheses could be used in non-oncological conditions, such as posttraumatic critical size bone defects and non-union of severely comminuted or periprosthetic fractures. However, since megaprostheses replace the affected bone tissue instead of trying to achieve bone healing, it has been suggested that they can be considered as a treatment option only in extreme, appropriately selected cases, mostly in elderly osteoporotic patients with poor bone quality and/or in those with limited life expectancy [40]. Since our patient was only 22 years old and had good bone quality and healing potential, we decided to perform the salvage procedure although we knew that it was necessary to perform hip arthroplasty due to the ONFH. Specifically, our aim was to reconstitute bone stock that could facilitate femoral anchorage of the modular prosthesis stem thereby reduce the risk of femoral loosening, and to enable biological reinsertion of the abductor apparatus which could provide a better functional result. This approach seemed reasonable as the survival rate of modular stems over 10 years of follow-up was reported to be 99 % on average [41], [42], compared to 52 % – 73 % in patients operated on with magaprostheses [43], [44], [45], [46].

The Masquelet technique, also known as the ‘induced membrane technique’, has been used successfully in the management of bone defects of any etiology and any size. The procedure is applied mainly in large bone defects in forearm, tibia and femur [47], [48]. The Masquelet technique often relies on external fixation for stability. External fixation around the proximal femur is uncomfortable, poorly tolerated by most patients and related to complications of which pin-site infection is the most common. There is a paucity of literature focusing solely on the reconstruction of non- or malunions following fractures in the proximal femur. In one case study, the Masquelet procedure was reported in a 24-year-old man with osteomyelitis following to subtrochanteric fractures operated on with a plate [49]. In our patient, only superficial wound infection, but not osteomyelitis, was observed shortly after the first operation. Since the femur malunion and bone defect were not infected, we decided to perform the Masquelet operation and reconstruction of the bone axis with the use of an intramedullary nail instead of external fixation. The stage two operation, concerning removal of the cement spacer and filling the defect with bone graft, was performed according to previous recommendations. There is. however. no evidence-based algorithm for the selection of the graft type or donor area. Since we could not harvest a sufficient amount of the cancellous bone from the iliac crest and a Reamer-Irrigator-Aspirator (RIA) system [31] was not available, we used a mixture of fresh autograft bone graft and freeze-dried morselized cancellous allograft in a ratio of 1:3, contrary to previously described proportions of 70 to 30% [50] or 3:1 [51]. However, due to the good bone healing potential, a good bony union was achieved 13 months after stage two surgery (Fig. 3).

When performing the THA, we decided to use a modular femoral stem. Such a choice enabled us to restore the center of rotation, achieve soft-tissue balancing and equalize leg length. With respect to the narrow medullary canal however, we had to select the narrowest stem, but even then it was technically demanding. The patient was mobilized quickly after the operation. She improved remarkably already during the first months following the THA. She accomplished over 90 degrees of flexion and good walking ability without limp within 6 months. Her HOOS scores were close to the functional recovery values, matching female and 18–34 years age frame, as defined in the earlier study based on the Swedish Reference data [52].

## Conclusion

We have demonstrated how we have managed a malunion in the proximal femur and ONFH following an AO/OTA 31-A3 fracture. The Masquelet technique was performed and allowed to reconstruct the proximal femur defect, enabling us to insert the stem of a hip prosthesis which was necessary to replace the damaged hip joint. The long-lasting, multi-stage salvage treatment was effective and eventually successful. It could have been, however, avoided if appropriate primary treatment had been applied.

## Consent for publication

A verbal and informed written consent was obtained to use the patient's clinical data and images to publish this case report; no identification of the patient's identity is present neither in the manuscript nor in the images. The authors confirm that this work was performed in accordance with The Code of Ethics of the World Medical Association (Declaration of Helsinki).

## Availability of data and material

All the data regarding the presented case are included within the article.

## Funding

We did not receive any specific grant from funding agencies in the public, commercial, or not-for-profit sectors.

## CRediT authorship contribution statement

P.T.P. carried out the idea and performed the surgery, P.T.P. and K.S. carried out data acquisition and assessment. P.T.P. did the literature search, drafted the manuscript and designed the figures. All authors participated in operations. All authors read and approved the final manuscript.

## Declaration of competing interest

No conflict of interest, financial or other, exists.
